# UNC5H4-induced apoptosis in non-small cell lung cancer is not dependent on p53 status only

**DOI:** 10.3892/ol.2013.1571

**Published:** 2013-09-09

**Authors:** ZHONG-HAI ZHAO, LE LIN, A. LIANG, HONG-QIU LI, YUE ZHU

**Affiliations:** 1 Department of Orthopedics, The First Affiliated Hospital of China Medical University, Shenyang, Liaoning 110004, P.R. China; 2 Department of Orthopedics, The Affiliated Zhong-Shan Hospital of Dalian University, Dalian, Liaoning 116011, P.R. China

**Keywords:** UNC5H4, p53, apoptosis, non-small cell lung cancer, X-ray

## Abstract

The aim of the present study was to investigate the expression profile and prognostic significance of uncoordinated 5 homolog 4 (UNC5H4) in patients with lung cancer and to evaluate whether UNC5H4 expression may serve as an index for radiosensitivity. UNC5H4 and p53 expression levels were detected by immunohistochemistry, apoptosis was determined by a terminal deoxynucleotidyl transferase-mediated dUTP nick end labeling assay and caspase 3 activation was determined by western blotting. The results showed that UNC5H4 expression was largely located in the membrane of the normal bronchial epithelium, but absent in the membranous regions or ectopic cytoplasm of 80/130 (61.5%) non-small cell lung cancer (NSCLC) tissue samples. Abnormal UNC5H4 expression was demonstrated to correlate with the degree of differentiation (P=0.015), TNM staging (P=0.037). Cytoplasmic UNC5H4 expression was shown to correlate negatively with p53 mutant type (mt) expression (r=−0.270; P=0.002) and positively with the apoptotic index (r=0.254; P=0.004). The statistical analyses indicated that the prognosis of patients with normal UNC5H4 expression was improved compared with that of patients with abnormal UNC5H4 expression, however, no significant difference was identified (P=0.125). Exposure of NSCLC tissue samples to X-radiation increased UNC5H4 expression and caspase 3 activity significantly, irrespective of p53 mutation status. In conclusion, these results indicate that X-rays induce apoptosis via the p53 pathway, and when this pathway is compromised, an additional pathway is utilized.

## Introduction

UNC5H1-4 are the mammalian homologs of *Caenorhabditis elegans* uncoordinated 5 (UNC5). These homologs have two immunoglobulin and two thrombospondin type 1 domains in their extracellular regions, as well as a deleted in colorectal cancer (DCC) and C terminal death domains in their cytoplasmic regions (1,2).

The mammalian UNC5H family is composed of UNC5H1-4 (2,3) and among these, human UNC5H4 cDNA encodes a 948 aa residue with a putative 14 aa signal peptide and 359 aa extracellular domain. The extracellular domain of human UNC5H4 shares ~97 and 66% aa sequence identity with mouse UNC5H4 and human UNC5H3, respectively. Similar to the other UNC5H family members, UNC5H4 contains a canonical caspase cleavage site in its cytoplasmic region, and forced expression of UNC5H4 has been reported to result in cell apoptosis (4,5). In addition, Wang *et al* previously reported that UNC5H4-mediated apoptosis is dependent on p53 status (6).

The hyposensitivity of non-small cell lung cancer (NSCLC) to radiation is a major cause of failure in radiotherapy. By increasing the radiation dose, the local control of tumor development may be improved, however, it may also result in various side-effects (7). Thus, the therapeutic efficiency of X-radiation in NSCLC treatment may be improved by increasing the sensitivity of low-sensitivity NSCLC tissue to formulate a personalized and reasonable chemoradiotherapy program for patients with NSCLC of varying sensitivity levels. Radiobiology has demonstrated that cell apoptosis is an important index of radiosensitivity since radiation induces tumor cell apoptosis by activating p53 and additional signal pathways (8,9). Therefore, an analysis of the correlation between UNC5H4 and p53 expression and apoptosis in lung cancer tissue through exposure to clinical doses of radiation under *in vitro* culture conditions may be beneficial.

To date, there have been no clinicopathological studies that have analyzed the correlation between UNC5H4 expression and human lung cancer. In the present study, the pattern and prognostic significance of UNC5H4 expression were examined in patients with lung cancer. The gene expression profile of UNC5H4 in NSCLC was measured and the correlation between UNC5H4 expression and patient pathological features and clinical outcome were analyzed. In addition, the effects of UNC5H4 and p53 expression on NSCLC prognosis were analyzed from follow-up data. By exposing fresh NSCLC tissues to X-rays, the changes in UNC5H4, p53 wild type (wt), p53 mutant type (mt) and, in particular, caspase 3 expression, were observed in order to evaluate whether UNC5H4 expression may represent an index for the radiosensitivity of NSCLC patients.

## Materials and methods

### Tissue samples and tissue microarray (TMA) development

Tumor specimens, including NSCLC and paired non-tumor tissues (obtained >5 cm from the primary tumor edge), were extracted from 130 patients with NSCLC between 2000 and 2005 following surgical resection at the Hunan Province People’s Hospital (Changsha, China). This study was approved by the local institutional review board at China Medical University (Shenyang, China). Written informed consent was obtained from the patients. The patients had not received radiotherapy, chemotherapy or immunotherapy prior to the tumor excision. The patient ages at the time of surgery ranged between 35 and 77 years old, with an average age of 54.38 years old. According to the TNM staging revised by the International Union Against Cancer in 2007 (10) and the classification of the World Health Organization (11), the patients with primary lung cancer were classified into two groups, including 69 squamous cell carcinomas (15, 21, 30 and 3 cases at stages I, II, III and IV, respectively) and 61 adenocarcinomas (19, 8, 33 and 1 cases at stages I, II, III and IV, respectively). The complete follow-up records of 70/130 patients and the lymph node metastases of 35/130 patients were available.

TMAs were constructed from formalin-fixed and paraffin-embedded tumor tissues by selecting viable and representative regions enriched in tumor cells. Core samples were removed (diameter, 1.6 mm) from each tumor and rearranged on empty paraffin-blocks using a manual TMA device (MTA-II; Beecher Instruments, Inc., Sun Prairie, WI, USA).

### Immunohistochemical staining

Immunohistochemical staining was performed using the streptavidin-peroxidase system (Ultrasensitive; MaiXin Technology Co., Ltd., Guangdong, China) according to the manufacturer’s instructions. Briefly, the TMAs were deparaffinized in xylene and rehydrated with graded alcohol solutions. Each specimen was incubated with 3% hydrogen peroxide, followed by incubation overnight at 4°C with primary antibodies against UNC5H4 (1:100; sc-67978; Santa Cruz Biotechnology Inc., Santa Cruz, CA, USA), p53 mt (1:200; DO-7; Thermo Fisher Scientific Inc., Waltham, MA, USA) and p53 wt (1:200; Ab-5; Calbiochem-Merck Co., Darmstadt, Germany). Biotinylated immunoglobulin G (IgG) was used as a secondary antibody (Cell Signaling Technology, Inc., Danvers, MA, USA). The slides were washed with phosphate-buffered saline and the antibody reaction was visualized using a fresh substrate solution containing diaminobenzidine. The TMAs were counter-stained with hematoxylin and then the immunostained TMAs were independently reviewed by two pathologists. Sections were defined as negative or positive according to the positive staining rate for p53 as follows: <5%, negative; and >5%, positive (12). For UNC5H4, normal expression was defined by >90% cell membrane staining of the tumor cells. The abnormal expression of UNC5H4 was defined by <90% cell membrane staining (reduced membranous expression) and >10% cytoplasmic staining (ectopic cytoplasmic expression) of the tumor cells.

### NSCLC tissue exposure to X-rays

Fresh tissues were isolated from 20 NSCLC patients, including 9 squamous cell carcinomas and 11 adenocarcinomas (5 well-, 13 moderately- and 2 poorly-differentiated tissues), and normal lung tissue was obtained from the resected lung of an NSCLC patient. The samples were immediately sectioned into 4×4×1-mm blocks and cultured in Dulbecco’s modified Eagle’s medium (DMEM; Gibco-BRL, Carlsbad, CA, USA) with 10% fetal calf serum at 37°C. Tissues were exposed to 1 Gy irradiation with 6 MeV X-rays at 37°C using a linear accelerator (PRIMUS; Siemens, Munich, Germany), as described previously (13). Exposed tissues were cultured in DMEM for 5 h at 37°C in a 5% CO_2_ incubator and then harvested.

### Apoptosis assay

Apoptosis was detected in the tissues by terminal deoxynucleotidyl transferase-mediated dUTP nick end labeling (TUNEL) assays using an *In Situ* Cell Death Detection kit (Roche Diagnostics GmbH, Mannheim, Germany), according to the manufacturer’s instructions.

Sections were dewaxed in xylene, taken through gradient ethanol (100, 95, 90, 80 and70%, each for 10 min), diluted in double distilled water and incubated with 30 ml/l hydrogen peroxide in methanol for 30 min. The sections were then washed with PBS, incubated in dialysate solution for 2 min on ice and incubated with TUNEL reaction mixture at 37°C for 30 min. Next, the samples were washed with PBS, incubated with converter peroxidase at 37°C for 1 h and stained with 3,3′-diaminobenzidine tetrahydrochloride. A negative control was performed in parallel using all reagents, with the exception of terminal transferase. The nuclei of positive cells were stained brown and detected under light microscopy. A total of 10 optical fields of 500–1,000 cells were counted in each slide under high power microscopy (magnification, ×400). The apoptosis index (AI) was calculated as the percentage of positive cells in 1,000 cells and then divided into two groups; low AI, ≤5% and high AI, >5%.

### Western blot analysis

Tissue lysate (50 mg) was resolved by 12% sodium dodecyl sulfate-polyacrylamide gel electrophoresis and transferred onto polyvinylidene difluoride membranes (Sigma-Aldrich, St Louis, MO, USA). The membranes were blocked with 3% fetal bovine serum and incubated with antibodies against UNC5H4 (1:200; sc-14029, Santa Cruz Biotechnology Inc.), p53 mt (1:200), p53 wt (1:200; Ab-5; Calbiochem-Merck Co.), caspase 3 (1:400; ab7980; Abcam, Cambridge, UK) and β-actin (1:200) overnight at 4°C. The samples were then incubated with horseradish peroxidase-conjugated IgG secondary antibody and bands were visualized by enhanced chemiluminescence (Thermo Fisher Scientific Inc.).

### Reverse transcription-PCR

Total RNA was extracted from cell tissue using TRIzol reagent (Invitrogen Life Technologies, Carlsbad, CA, USA) according to the manufacturer’s instructions. A reverse transcription-PCR analysis was performed using a Takara RNA PCR kit (AMV) version 3.0 (Takara Bio, Inc., Shiga, Japan) and the following primers: UNC5H4 sense, 5′-ACCGAAAGTACATTCTTGATAAC-3′ and antisense, 5′-TCCATACCTGAACTCTCTGC-3′; and β-actin sense, 5′-AGAGCTACGAGCTGCCTGAC-3′ and antisense, 5′-AGT ACTTGCGCTCAGGAGGA-3′. The PCR conditions were set at 94°C for 4 min, 35 cycles of 94°C for 1 min, 52°C (UNC5H4) and 55°C (β-actin) for 30 sec, 72°C for 30 sec and then 72°C for 10 min. The products were resolved by 1% agarose gel and the bands were visualized by ethidium bromide staining. Densitometric analyses of the bands were performed using a EC3 Imaging System (UVP LLC, Upland, CA, USA).

### Statistical analysis

Data are presented as the mean ± SD. SPSS version 13.0 (SPSS, Inc., Chicago, IL, USA) was used for all the analyses. The correlation between the percentage of the UNC5H4 and p53 mt-positive staining rates and the AI was analyzed using Spearman’s rank correlation coefficient and the correlation between UNC5H4 and p53 mt expression and the related clinical and pathological factors was analyzed using the χ^2^ test. Survival rate analyses were performed using the correlation between UNC5H4 and p53 mt expression and the prognosis of patients by the Kaplan-Meier method (log-rank test). Differences in the expression of UNC5H4 and the activation of caspase 3 in the lung cancer tissues and cells were determined by Student’s t-test. P<0.05 was considered to indicate a statistically significant difference.

## Results

### Correlation between UNC5H4 and p53 mt expression and the AI

**Figure 1:**
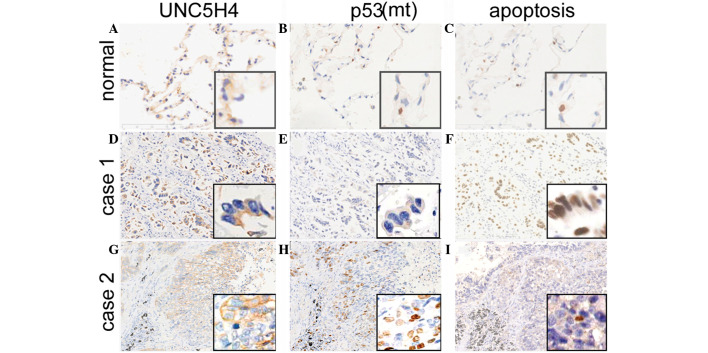
Correlation between p53 mt and UNC5H4 expression and apoptosis in normal alveolar epithelial cells and NSCLC tissue. (A) Cytoplasmic UNC5H4 and (B) p53 mt expression and (C) apoptosis in normal alveolar epithelial cells. Ectopic cytoplasmic expression of (D) UNC5H4 was shown to positively correlate with (F) a high AI. (E) Nuclear p53 mt-negative in poorly-differentiated adenocarcinoma tissue. (G) Low cytoplasmic UNC5H4 expression was identified in membranous regions in well-differentiated squamous cell carcinoma with (H) high nuclear p53 mt expression and (I) a low AI. As defined by immunohistochemical S-P and TUNEL (magnification, ×400). NSCLC, non-small cell lung cancer; S-P, streptavidin-peroxidase; TUNEL, terminal deoxynucleotidyl transferase-mediated dUTP nick end labeling; mt, mutant type; AI, apoptosis index; UNC5H4, uncoordinated 5 homolog 4.

The expression of UNC5H4 occurred in the cell membrane of the normal bronchial epithelium and particularly in the cytoplasm of the alveolar epithelial cells (Fig. 1A). Normal or preserved membranous UNC5H4 expression was identified in 50/130 (38.5%) patients, while 80/130 (61.5%) patients were identified with abnormal UNC5H4 expression, including absent membranous expression with ectopic cytoplasmic expression (Fig. 1D) and reduced membranous expression with ectopic cytoplasmic expression (Fig. 1G). In addition, abnormal UNC5H4 expression was shown to correlate with the degree of differentiation (P=0.015) and TNM staging (P=0.037), as shown in Table I. The abnormal expression rates for the well- and moderately-differentiated cells (47.9%) were significantly higher compared with those of the poorly-differentiated cells (69.5%; P=0.015), and significantly higher for TNM stages III and IV (70.1%) compared with stages I and II (52.4%; P=0.037). In addition, the abnormal expression rate for patients with lymph node metastasis (69.8%) was significantly higher when compared with that of patients with no lymph node metastasis (53.7%; P=0.059). No significant differences were identified among patient gender, age, histological type or lymphatic metastasis.

The p53 mt-positive staining rate in the NSCLC tissue was 39.2% (51/130 patients) and the correlation between positive expression and the clinicopathological characteristics of the patients with NSCLC are summarized in Table I. The expression of p53 mt was revealed to correlate significantly with TNM staging and lymph node metastasis (P<0.05). Cytoplasmic UNC5H4 expression was revealed to correlate negatively with p53 mt expression (r=−0.270; P=0.002) and positively with the AI (r=0.254; P=0.004). In addition, the expression of p53 mt was shown to correlate negatively with the AI (r=−0.190; P=0.09) ( Fig. 1).

### Effect of abnormal UNC5H4 and p53 mt expression on NSCLC patient prognosis

**Figure 2:**
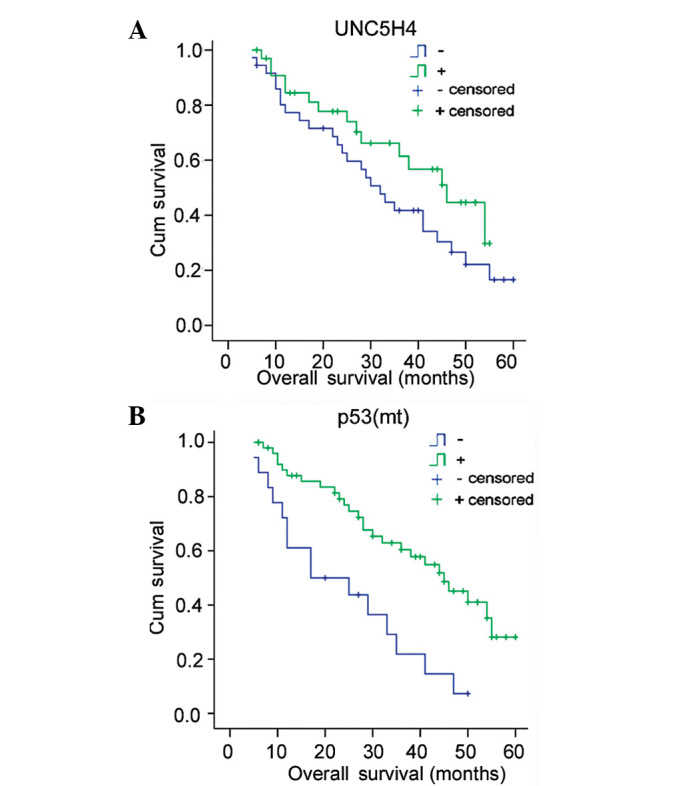
Correlation between (A) UNC5H4 and (B) p53 mt expression and the survival rate of 70 NSCLC patients from the day of surgery. mt, mutant type; NSCLC, non-small cell lung cancer. UNC5H4, uncoordinated 5 homolog 4.

The post-surgical survival time ranged between 5 and 60 months, the average survival time was 36.6±2.4 months and the median survival time was 38.0±5.0 months in 70 NSCLC patients. The Kaplan-Meier method (log-rank test) was used to analyze life span. The average and median survival times for each group were as follows: i) Abnormal UNC5H4 expression, 33.2±3.2 and 32.0±3.6 months; ii) normal UNC5H4-positive, 39.22±3.2 and 46.0±7.2 months; iii) p53 mt-negative, 40.9±2.7 and 45.0±5.7 months; and iv) p53 mt-positive, 24.10±3.6 and 17.0±8.8 months, respectively. The statistical analyses indicated that the prognosis of the patients with normal UNC5H4 expression was improved when compared with that of patients with abnormal UNC5H4 expression, however, no significant difference was identified (P=0.125). The prognosis of the p53 mt-negative patients was significantly improved when compared with that of the p53-positive patients (P=0.001) (Fig. 2).

A Cox regression analysis was used to evaluate the significance of abnormal UNC5H4 and p53 mt expression as prognostic factors (Table II). Age, gender, histology, degree of differentiation, TNM stage, lymph node metastasis, abnormal UNC5H4 expression and p53 mt-positive expression were analyzed in 70 patients, revealing p53 mt expression (P=0.006), lymph node metastasis (P=0.001), tumor stage (P=0.002) and degree of differentiation (P=0.044) as independent prognostic factors. However, abnormal UNC5H4 expression was not identified as an independent prognostic factor (P=0.073).

### Effect of X-ray exposure on UNC5H4 expression and apoptosis in NSCLC tissue

**Figure 3:**
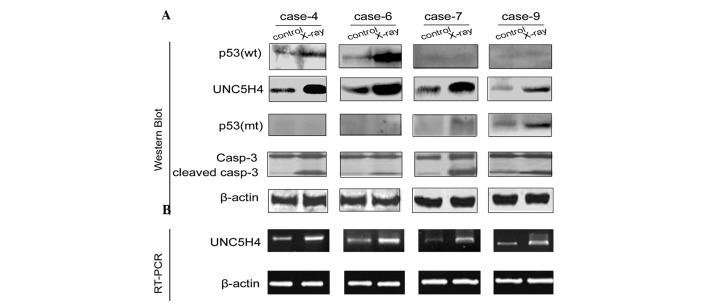
(A) Western blotting of UNC5H4 and wild type (wt) and mutant type (mt) p53 expression and caspase 3 activation (cleaved caspase 3). (B) Reverse transcription-PCR analysis of UNC5H4 expression in NSCLC tissue following X-ray exposure. NSCLC, non-small cell lung cancer; UNC5H4, uncoordinated 5 homolog 4.

NSCLC tissue was exposed to 1 Gy of X-radiation and cultured for 5 h. A radiation-induced increase in the UNC5H4 mRNA and protein levels was identified in 13/20 cases, including 6 squamous cell carcinoma and 7 adenocarcinoma cases (4 well-, 8 moderately- and 1 poorly-differentiated; Fig. 3). In addition, the activation of caspase 3 was also significantly increased in these cases, indicating that UNC5H4 may play a role in X-ray-induced apoptosis.

**Figure 4:**
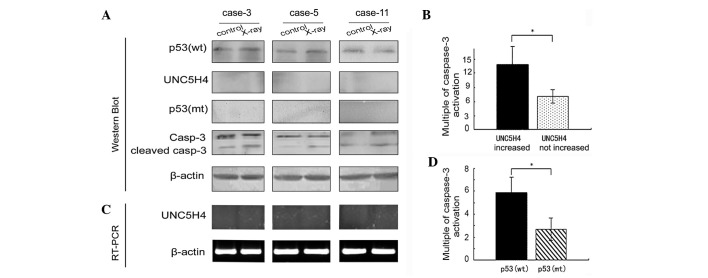
(A) Western blotting and (C) reverse transcription-PCR analysis of UNC5H4 and p53 wt and mt expression and caspase 3 activation (cleaved caspase 3) in NSCLC tissue following X-ray exposure in UNC5H4-negative/low cases. (B) Caspase 3 expression of all 20 cases studied. (D) Of the 13/20 cases that exhibited increased expression in (B) 6/13 and 7/13 were p53 mt-positive and -negative, respectively. ^*^P<0.05. wt, wild type; mt, mutant type; UNC5H4, uncoordinated 5 homolog 4.

Notably, changes were not detected in the expression of UNC5H4 in the remaining 7/20 cases, including 3 squamous cell carcinoma and 4 adenocarcinoma cases (1 well-, 5 moderately- and 1 poorly-differentiated), however, significant increases in caspase 3 protein activation levels were observed (Fig. 4A and B), but were shown to be significantly lower when compared with those of the 13 NSCLC cases with increased UNC5H4 expression (P<0.05; Fig. 4C). In addition, 6/13 cases demonstrating increased expression of UNC5H4 and p53 mt exhibited significantly lower caspase 3 activation levels compared with the p53 wt cells (P<0.05; Fig. 4D).

## Discussion

It has been previously reported that all four members of the UNC5H family exhibit proapoptotic activity (5,14) and share a similar canonical caspase cleavage site in their cytoplasmic region (13), which is important for the induction of apoptosis. Since the caspase-mediated cleavage of UNC5H is required for cell death induction, in the current study, caspase 3 expression was detected to investigate the induction of apoptosis. In contrast to UNC5H1-3, little is known with regard to the functional significance of UNC5H4. More recent studies have identified that the UNC5H4-mediated induction of apoptosis is dependent on p53 status (6). However, the correlation between UNC5H4 expression and apoptosis via the p53 pathway remains unclear in lung cancer.

Fresh tissues obtained from 20 NSCLC patients were exposed to clinical doses of X-rays under *in vitro* culture conditions. Notably, the exposure of NSCLC tissue to X-rays leads to the concurrent upregulation of UNC5H4 expression and caspase 3 activation. Therefore, NSCLC tissue with high UNC5H4 expression may be sensitive to radiation and highlight a new basis for a radiosensitive indicator for NSCLC patients, allowing for an improved and individualized dosing program. In addition, the results indicated that UNC5H4 upregulation is independent of p53 status in NSCLC tissue since UNC5H4 is a direct transcriptional target of p53 and UNC5H4-mediated apoptosis is regulated in a p53-dependent manner.

Therefore, the present study further investigated the correlation between UNC5H4 and p53 expression and apoptosis, as well as the correlation between UNC5H4 expression and the clinicopathological characteristics of NSCLC. UNC5H4 and p53 expression and apoptosis were determined in lung cancer tissue obtained from 130 NSCLC patients at the time of treatment. UNC5H4 expression was shown to correlate significantly with the degree of differentiation, TNM staging, lymphatic metastasis state and prognosis of NSCLC. The prognosis was significantly improved in patients with NSCLC tissues expressing high levels of UNC5H4 compared with patients whose NSCLC tissues expressed low levels. Studies have also indicated that UNC5H4-mediated, radiation-induced apoptosis correlates with p53 status and that p53 is a tumor suppressor capable of inducing cell cycle arrest and apoptosis (15–17). In the present study, UNC5H4 expression was demonstrated to positively correlate with radiation-induced AI and negatively correlate with p53 mt expression, indicating that UNC5H4-mediated apoptosis requires p53. However, a subset of cases that responded to radiotherapy were also shown to express p53 mt. UNC5H4 expression and caspase 3 activation are upregulated following X-ray exposure, indicating that UNC5H4 may induce apoptosis independently of p53. In conclusion, the results of the present study indicate that X-rays induce apoptosis via an additional pathway when the p53 pathway is compromised. Further analysis and identification of this pathway must be performed in future studies.

## Figures and Tables

**Table I:** Clinical and histological features of 130 patients with lung cancer.

		UNC5H4 expression, n			p53 expression, n		
					
Variables	Patients, n	Abnormal	Normal	P-value^a^	Positive	Negative	P value^a^
n	130	80	50		51	79	
Age, years							
≤55	56	37	19	0.355	25	31	0.511
>55	74	43	31		26	48	
Gender							
Male	66	42	24	0.618	23	43	0.299
Female	64	38	26		28	36	
Stage							
I/II	63	33	30	0.037	17	46	0.006
III/IV	67	47	20		34	33	
Histology							
Squamous cell carcinoma	69	46	23	0.201	24	45	0.269
Adenocarcinoma	61	34	27		27	34	
Differentiation							
Well, moderate	48	23	25	0.015	24	24	0.054
Poor	82	57	25		27	55	
Lymph node metastasis							
Yes	63	44	19	0.059	35	28	0.000
No	67	36	31		16	51	

a P-values were obtained by χ^2^ test (two-sided).

UNC5H4, uncoordinated 5 homolog 4.

**Table II:** Cox regression model for the predicted survival of 70 patients with lung cancer.

Factor	Risk	95% CI	P-value
Age (<50years)	0.672	0.336–1.436	0.151
Male gender	0.750	0.424–1.653	0.403
Histology (adenocarcinoma)	1.335	0.312–1.421	0.372
Degree of differentiation (Poor)	2.847	1.362–3.344	0.044
TNM stage (III/IV)	3.710	1.264–6.735	0.002
Lymphatic metastasis	4.014	1.351–7.811	0.001
Positive p53 mt expression	0.335	0.412–0.723	0.006
Abnormal UNC5H4 expression	0.302	0.459–1.506	0.073

CI, confidence interval; mt, mutant type; UNC5H4, uncoordinated 5 homolog 4.
